# A method for the production of D-tagatose using a recombinant *Pichia pastoris* strain secreting β-D-galactosidase from *Arthrobacter chlorophenolicus* and a recombinant L-arabinose isomerase from *Arthrobacter* sp. 22c

**DOI:** 10.1186/1475-2859-11-113

**Published:** 2012-08-23

**Authors:** Marta Wanarska, Józef Kur

**Affiliations:** 1 Department of Microbiology, Chemical Faculty, Gdańsk University of Technology, Narutowicza 11/12, 80-233, Gdańsk, Poland; 2 BLIRT S.A., Trzy Lipy 3/1.38, 80-172, Gdańsk, Poland

## Abstract

**Background:**

D-Tagatose is a natural monosaccharide which can be used as a low-calorie sugar substitute in food, beverages and pharmaceutical products. It is also currently being tested as an anti-diabetic and obesity control drug. D-Tagatose is a rare sugar, but it can be manufactured by the chemical or enzymatic isomerization of D-galactose obtained by a β-D-galactosidase-catalyzed hydrolysis of milk sugar lactose and the separation of D-glucose and D-galactose. L-Arabinose isomerases catalyze *in vitro* the conversion of D-galactose to D-tagatose and are the most promising enzymes for the large-scale production of D-tagatose.

**Results:**

In this study, the *araA* gene from psychrotolerant Antarctic bacterium *Arthrobacter* sp. 22c was isolated, cloned and expressed in *Escherichia coli*. The active form of recombinant *Arthrobacter* sp. 22c L-arabinose isomerase consists of six subunits with a combined molecular weight of approximately 335 kDa. The maximum activity of this enzyme towards D-galactose was determined as occurring at 52°C; however, it exhibited over 60% of maximum activity at 30°C. The recombinant *Arthrobacter* sp. 22c L-arabinose isomerase was optimally active at a broad pH range of 5 to 9. This enzyme is not dependent on divalent metal ions, since it was only marginally activated by Mg^2+^, Mn^2+^ or Ca^2+^ and slightly inhibited by Co^2+^ or Ni^2+^. The bioconversion yield of D-galactose to D-tagatose by the purified L-arabinose isomerase reached 30% after 36 h at 50°C. In this study, a recombinant *Pichia pastoris* yeast strain secreting β-D-galactosidase *Arthrobacter chlorophenolicus* was also constructed. During cultivation of this strain in a whey permeate, lactose was hydrolyzed and D-glucose was metabolized, whereas D-galactose was accumulated in the medium. Moreover, cultivation of the *P. pastoris* strain secreting β-D-galactosidase in a whey permeate supplemented with *Arthrobacter* sp. 22c L-arabinose isomerase resulted in a 90% yield of lactose hydrolysis, the complete utilization of D-glucose and a 30% conversion of D-galactose to D-tagatose.

**Conclusions:**

The method developed for the simultaneous hydrolysis of lactose, utilization of D-glucose and isomerization of D-galactose using a *P. pastoris* strain secreting β-D-galactosidase and recombinant L-arabinose isomerase seems to offer an interesting alternative for the production of D-tagatose from lactose-containing feedstock.

## Background

D-Tagatose is a rare natural ketohexose, a C-4 epimer of D-fructose and an isomer of aldohexose D-galactose. It occurs naturally as a component of the gum exudate of the tropical tree *Sterculia setigera*[1]. D-Tagatose is also found in small amounts in dairy products such as in-container sterilized cow’s milk [2], UHT lactose-hydrolyzed milk [3], and various cheeses and yogurts [4]. This monosaccharide is readily soluble in water (58% w/w at 21°C), stable at a pH range of 2 to 7, and has sweet taste similar to that of sucrose. D-Tagatose is 92% as sweet as sucrose, but its caloric value is much lower, at 1.5 kcal/g for humans, which is 37.5% of the caloric value of sucrose (4 kcal/g) [5]. Moreover, D-tagatose has a greater sweetness and lower caloric value than many mono- and disaccharides, namely D-glucose, D-galactose, lactose and maltose, and most of the sugar alcohols widely used as low-calorie sweeteners [6]. Since D-tagatose has received GRAS (Generally Recognized as Safe) approval from the U.S. Food and Drug Administration, it can be used in confectionery, beverages and dietary products as a low-calorie sweetener. D-Tagatose has been also approved as a food ingredient in Australia, New Zealand, Brazil, Korea, Japan and the European Union. Furthermore, D-tagatose has numerous health and medical benefits, including prebiotic, antioxidant and tooth-friendly properties. It is also safe for diabetics [5]. According to the WHO (World Health Organization), 346 million people worldwide have diabetes, 90% of whom suffer from type 2. D-Tagatose is currently being tested as a potentially important new drug for treating type 2 diabetes and obesity [7].

D-Tagatose can be produced by the oxidation of D-galactitol using microorganisms like *Arthrobacter globiformis*[8], *Mycobacterium smegmatis*[9], *Enterobacter agglomerans*[10] or *Gluconobacter oxydans*[11,12]. The maximum yield from biotransformation has been reported as being 92% [10]. However, this process cannot be used for large-scale D-tagatose production because of the high cost and unavailability of D-galactitol as a raw material. A method for producing D-tagatose by the bioconversion D-psicose using various strains of *Mucoraceae* fungi has also been described [13]. D-Psicose is a rare sugar, but it can be produced by the epimerization of relatively cheap raw material D-fructose using D-tagatose 3-epimerase from *Pseudomonas cichorii*[14] or *Rhodobacter sphaeroides*[15], as well as D-psicose 3-epimerase from *Agrobacterium tumefaciens*[16]. However, the method for producing D-tagatose from D-fructose via D-psicose seems to have little potential for commercial application.

Isomerization of D-galactose is an economically viable method for D-tagatose production. D-Galactose can be isomerized chemically or enzymatically. The chemical method for D-tagatose production was developed and patented by Biospherics Incorporated (USA). According to the patent, D-galactose can be isomerized to D-tagatose with calcium hydroxide and in the presence of calcium chloride as a catalyst. Under strongly alkaline conditions, and at a relatively low temperature, calcium hydroxide forms an insoluble complex with D-tagatose. Treatment of the suspension with acid, preferably carbon dioxide, liberates D-tagatose from the complex. The insoluble calcium salt is then removed by filtration. The remaining ions are eliminated by ion exchange chromatography. The crude syrup is then concentrated, and the pure D-tagatose is recovered by crystallization [17]. The chemical method does, however, have some disadvantages, such as by-product and chemical waste formation. The process is also energy-consuming, since it requires intensive cooling of the reaction mixture.

The enzymatic method of producing D-tagatose from D-galactose uses L-arabinose isomerase as a biocatalyst. *In vivo* L-arabinose isomerase (EC 5.3.1.4) catalyzes the conversion of L-arabinose to L-ribulose, which is the first step of this aldopentose metabolism in bacteria with arabinose operon. This enzyme is also able to isomerize D-galactose to D-tagatose, *in vitro*. Several microorganisms have been reported as sources of L-arabinose isomerases. Active enzymes towards D-galactose were isolated from mesophiles such as *Escherichia coli*[18], *Bacillus halodurans*[19], *Lactobacillus plantarum*[20] and *Lactobacillus fermentum*[21]; thermophiles such as *Geobacillus stearothermophilus*[19], *Geobacillus thermodenitrificans*[22], *Bacillus stearothermophilus* US100 [23], *Bacillus stearothermophilus* IAM11001 [24], *Alicyclobacillus acidocaldarius*[25], *Anoxybacillus flavithermus*[26], *Thermus* sp. IM6501 [27], *Acidothermus cellulolytics*[28] and *Thermoanaerobacter mathranii*[29]; and hyperthermophiles like *Thermotoga neapolitana*[30] and *Thermotoga maritima*[31]. An L-arabinose isomerase from psychrotolerant bacterium *Lactobacillus sakei* 23 K has also recently been described [32]. Most of these enzymes are optimally active at elevated temperatures ranging from 60 to 95°C and at a pH of above 7; only L-arabinose isomerase from *L. sakei* 23 K exhibits optimum activity at low temperatures ranging from 30 to 40°C and at a pH range of 5 to 7. Moreover, almost all the L-arabinose isomerases characterized require divalent metal ions such as Mn^2+^ or Co^2+^ for high activity and stability, with L-AI from mesophilic bacterium *B. halodurans* being the only one which is not dependent on them. However, the industrial application of L-arabinose isomerase requires an acidic pH and relatively low temperatures in order to reduce by-product formation and save energy [25]. Furthermore, the isomerization reaction should be conducted without the addition of divalent metal ions, especially Co^2+^ ions, as these are unacceptable in food applications.

Both the chemical and enzymatic methods for producing D-tagatose use D-galactose as a substrate. D-Galactose can be obtained from the hydrolysis of pure lactose or lactose-containing materials such as whey or whey permeate, both of them plentiful and inexpensive dairy industry by-products. In most of the processes described, lactose is enzymatically hydrolyzed using commercially available β-D-galactosidases from *Aspergillus* sp. [17,33,34]. The mixture of D-glucose and D-galactose is then separated by means of chromatography and the pure D-galactose is isomerized [17,34]. D-Glucose and D-galactose can be also separated by selective fermentation of D-glucose using *Saccharomyces cerevisiae* yeast [33]. However, this process should be strictly monitored and completed directly after D-glucose utilization, since *S. cerevisiae* is also able to ferment D-galactose.

For these reasons, new, simple methods of D-tagatose production are still sought. The present study was conducted with a view to developing a single-step method for producing D-tagatose directly from whey permeate. To this end, a recombinant *Pichia pastoris* yeast strain secreting β-D-galactosidase from *Arthrobacter chlorophenolicus* was constructed and a recombinant L-arabinose isomerase from psychrotolerant bacterium *Arthrobacter* sp. 22c was obtained and characterized. The combination of the recombinant yeast strain and the enzyme in one reaction mixture containing whey permeate resulted in the simultaneous hydrolysis of lactose, the utilization of D-glucose and the isomerization of D-galactose to D-tagatose.

## Results

### Selection and identification of strain 22c, producing L-arabinose isomerase

In order to select a microorganism producing L-arabinose isomerase with high activity at a low temperature, twenty bacterial strains previously isolated from Antarctic soil samples were grown, in an LBS medium containing L-arabinose, for the induction of *araA* gene expression. The enzyme activity in the cell extracts was then determined at 25°C and pH 7.0, using D-galactose as a substrate. The cell extracts of only three isolates named 22c, 32b and 32c exhibited L-arabinose isomerase activity. The bioconversion yields of D-galactose to D-tagatose obtained after 24 h were 2.75, 1.24 and 1.28% for cell extracts of strains 22c, 32b and 32c, respectively. The bacterial strain named 22c was selected for further study because of the highest L-arabinose isomerase activity. This isolate also exhibited amylase activity, but β-D-galactosidase, lipase/esterase and protease activities were absent. The optimum growth of strain 22c was observed at 24 to 26°C and no growth occurred at 37°C. Hence, isolate 22c can be categorized as a psychrotolerant microorganism.

The genus of strain 22c was assessed on the basis of the 16S ribosomal DNA gene sequence. An alignment of the 16S rDNA gene of isolate 22c (GenBank, accession no. JN642527) with the sequences available in the GenBank database demonstrated that strain 22c should be classified as an *Arthrobacter* sp.

### Cloning, expression and purification of *Arthrobacter* sp. 22c L-arabinose isomerase

**Figure 1:**
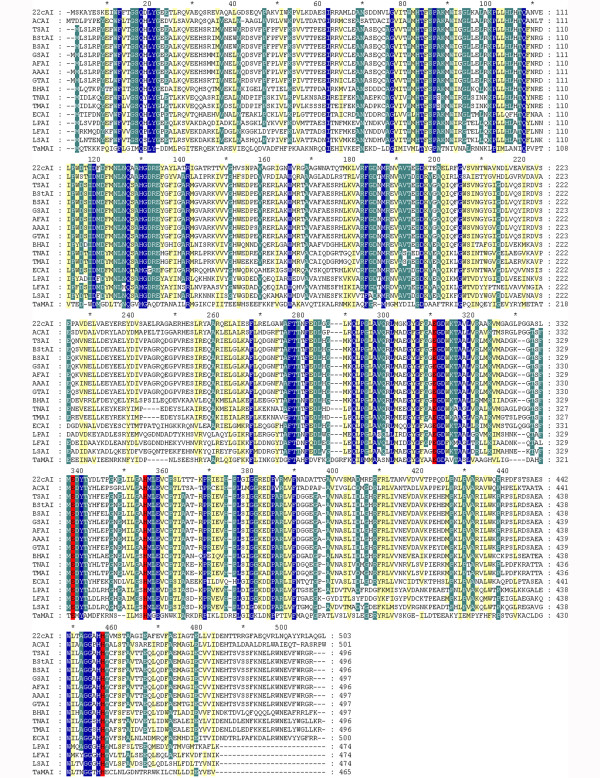
**Multiple sequence alignment of*Arthrobacter*sp. 22c L-arabinose isomerase with other L-arabinose isomerases.** 22cAI – *Arthrobacter* sp. 22c L-AI, ACAI – *Acidothermus cellulolytics* ATCC 43068 L-AI, TSAI – *Thermus* sp. IM6501 L-AI, BStAI – *Bacillus stearothermophilus* IAM 11001 L-AI, BSAI – *Bacillus stearothermophilus* US100 L-AI, GSAI – *Geobacillus stearothermophilus* L-AI, AFAI - *Anoxybacillus flavithermus* L-AI, AAAI – *Alicyclobacillus acidocaldarius* L-AI, GTAI – *Geobacillus thermodenitrificans* L-AI, BHAI – *Bacillus halodurans* L-AI, TNAI – *Thermotoga neapolitana* L-AI, TMAI – *Thermotoga maritima* L-AI, ECAI – *Escherichia coli* str. K-12 substr. W3110 L-AI, LPAI – *Lactobacillus plantarum* NC8 L-AI, LFAI – *Lactobacillus fermentum* CGMCC2921 L-AI, LSAI – *Lactobacillus sakei* 23 K L-AI, TaMAI – *Thermoanaerobacter mathranii* L-AI. Three levels of conserved residues are indicated by blue (100%), green (80%) and yellow (60%) backgrounds. Catalytic residues are shaded red. The alignment was performed using Clustalx 2.0.11 program.

The degenerated primers araDF and araAR were designed on the basis of the sequences and genetic organization of arabinose operons from *Arthrobacter* sp. FB24, *Arthrobacter aurescens* TC1 and *Arthrobacter chlorophenolicus* A6 (*araB*, *araD* and *araA*). A 1362 bp DNA fragment obtained by PCR using those primers contained 1134 bp fragment of putative *Arthrobacter* sp. 22c *araA* gene. The unknown part of the gene encoding L-arabinose isomerase adjacent to the known sequence was found using the GenomeWalker^TM^ Universal Kit (Clontech Laboratories, USA). Analysis of the sequences obtained revealed an open reading frame consisting of 1,512 bp, which encoded a 503 amino acid protein with a calculated molecular mass of 55,183 Da and a theoretical pI of 5.08 (ProtParam; ExPASy Proteomics Server). A homology search, performed using the BLAST (Basic Local Alignment Search Tool) at the NCBI (National Center for Biotechnology Information) displayed a 71, 70 and 69% identity with nucleotide sequences corresponding to the *araA* genes from *Arthrobacter* sp. FB24, *Arthrobacter chlorophenolicus* A6 and *Arthrobacter aurescens* TC1, respectively. The *Arthrobacter* sp. 22c *araA* gene product showed a 71.1% amino acid identity and an 89.1% similarity with L-arabinose isomerase from *Arthrobacter* sp. FB24, a 69.7% identity and an 88.2% similarity with L-AI from *A. chlorophenolicus* A6, and a 69.5% identity and an 88.6% similarity with L-AI from *A. aurescens* TC1. Furthermore, the *Arthrobacter* sp. 22c L-arabinose isomerase displayed a higher sequence identity with L-AIs from thermophilic bacteria such as *Acidothermus cellulolytics* ATCC 43068, *Bacillus stearothemophilus* US100, *Geobacillus stearothermophilus*, *Anoxybacillus flavithermus*, *Alicyclobacillus acidocaldarius*, *Thermus* sp. IM6501, *Geobacillus thermodenitrificans* and *Bacillus stearothermophilus* IAM 11001 than those from mesophiles like *Bacillus halodurans*, *Escherichia coli*, *Lactobacillus plantarum* NC8 and *Lactobacillus fermentum* CGMCC2921 or the psychrotolerant *Lactobacillus sakei* 23 K (Table 1). The multiple sequence alignment of *Arthrobacter* sp. 22c L-arabinose isomerase (22cAI) with its previously described counterparts from psychrotolerant, mesophilic, thermophilic and hyperhtermophilic microorganisms revealed some conserved regions and four essential catalytic residues, namely E307, E334, H351 and H451, corresponding to the E306, E333, H350 and H450 of *E. coli* L-arabinose isomerase and the E306, E331, H348 and H447 of *B. stearothermophilus* US100 L-AI (Figure 1) [35,36]. 

**Table 1:** **Comparison of the amino acid sequence of*Arthrobacter*sp. 22c L-arabinose isomerase with amino acid sequences of L-arabinose isomerases from various microorganisms**

**Organism**	**GenBank Accession No.**	**Identity (%)**	**Similarity (%)**
*Arthrobacter* sp. FB24	ABK01625	71.1	89.1
*Arthrobacter chlorophenolicus* A6	ACL38380	69.7	88.2
*Arthrobacter aurescens* TC1	ABM06985	69.5	88.6
*Acidothermus cellulolytics* ATCC 43068	ACZ67491	59.3	83.4
*Bacillus stearothermophilus* US100	CAI29261	54.6	81.3
*Geobacillus stearothermophilus*	AAD45718	54.6	81.3
*Anoxybacillus flavithermus*	AEE98871	54.6	81.1
*Alicyclobacillus acidocaldarius*	AAY68209	54.4	81.1
*Thermus* sp. IM6501	AAO72082	54.2	80.7
*Geobacillus thermodenitrificans*	AAQ72737	54.0	80.9
*Bacillus stearothermophilus* IAM 11001	ABY84698	53.8	80.7
*Thermotoga neapolitana*	AAK18729	52.8	78.7
*Thermotoga maritima*	AAD35365	52.0	77.6
*Bacillus halodurans*	BAB05592	50.3	79.3
*Escherichia coli* str. K-12 substr. W3110	BAB96631	50.0	79.1
*Lactobacillus sakei* 23 K	CAI56163	48.5	75.0
*Lactobacillus plantarum* NC8	CCC80517	48.3	74.4
*Lactobacillus fermentum* CGMCC2921	ADJ94948	43.7	71.9
*Thermoanaerobacter mathranii*	CAE46769	25.1	59.8

A homology search was performed using version 3 of the FASTA programme at the EBI (European Bioinformatics Institute).

**Figure 2:**
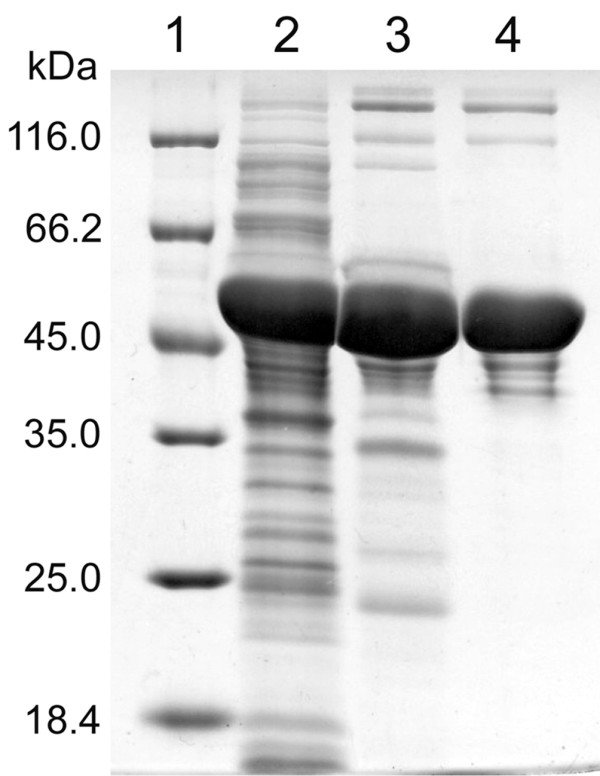
**SDS-PAGE analysis of the fractions obtained by expression and purification of*Arthrobacter*sp. 22c L-arabinose isomerase.** Lane 1 – Unstained Protein Molecular Weight Marker (Fermentas): 116, 66.2, 45, 35, 25, 18.4 and 14.4 kDa, lane 2 – cell extract of *E. coli* BL21(DE3)pLysS + pET30araA22c after *araA* gene expression, lane 3 – purified L-arabinose isomerase after ion exchange chromatography on Fractogel EMD DEAE column, lane 4 - purified L-arabinose isomerase after ion exchange chromatography on Fractogel EMD TMAE column.

In order to produce and investigate the biochemical properties of *Arthrobacter* sp. 22c L-arabinose isomerase, a recombinant pET30araA22c plasmid was constructed and used for the expression of the *Arthrobacter* sp. 22c *araA* gene in *E. coli* BL21(DE3)pLysS. The recombinant enzyme was purified by means of ion exchange chromatography using weak and strong anion exchangers. After purification and following sodium dodecyl sulfate-polyacrylamide gel electrophoresis and staining with Coomassie blue, large quantities of protein were observed migrating between 45 and 66 kDa (Figure 2, lane 4). This concurred with the molecular mass of *Arthrobacter* sp. 22c L-arabinose isomerase deduced from the nucleotide sequence of *araA* gene (55.2 kDa). The overexpression system and purification method applied were quite efficient, yielding 48 mg (Table 2) of *Arthrobacter* sp. 22c L-arabinose isomerase from 1 L of *E. coli* culture. The molecular mass of the native enzyme, estimated by gel filtration, was 335 kDa. Hence, it is assumed that the *Arthrobacter* sp. 22c L-arabinose isomerase is probably a hexameric protein.

**Table 2:** **Summary of the purification of recombinant*Arthrobacter*sp. 22c L-arabinose isomerase obtained from 1 L of*E. coli*BL21(DE3)pLysS culture**

**Purification step**	**Protein (mg)**	**Total activity (U)**	**Specific activity (U mg**^**-1**^**)**	**Purification (fold)**	**Recovery (%)**
Cell extract	490	12.76	0.026	1.0	100
Fractogel EMD DEAE	105	11.60	0.110	4.2	21
Fractogel EMD TMAE	48	11.14	0.232	8.9	10

The enzyme activity was measured with D-galactose as a substrate.

### Properties of *Arthrobacter* sp. 22c L-arabinose isomerase

**Figure 3:**
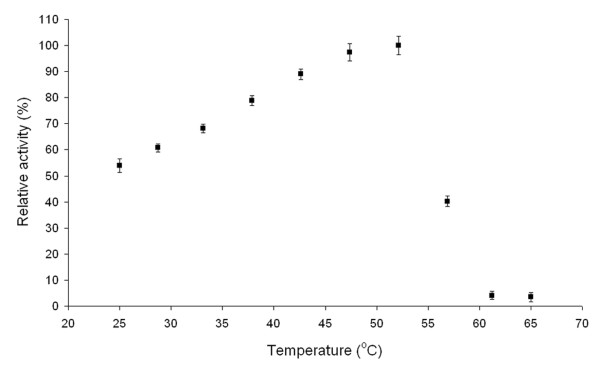
**Effect of temperature on activity of recombinant*Arthrobacter*sp. 22c L-arabinose isomerase.** Reaction mixtures containing 0.2 mg mL^-1^*Arthrobacter* sp. 22c L-arabinose isomerase and 4% (w/v) D-galactose in 20 mM potassium phosphate buffer pH 7.0 were incubated at temperatures from 25 to 65°C for 12 h.

Investigation into the effect of temperature on *Arthrobacter* sp. 22c L-arabinose isomerase showed that the highest isomerization activity with D-galactose as a substrate occurred at 52°C; however, the enzyme exhibited over 60% of maximum activity at 30°C (Figure 3).

The *Arthrobacter* sp. 22c L-arabinose isomerase showed high stability at 50°C and pH 7.0, since over 90% of its initial activity was retained after 72 h incubation. At 60 and 65°C, over 50% of initial activity was lost in incubations of 60 and 30 min, respectively. The enzyme was completely inactivated by 15 min at 70°C.

**Figure 4:**
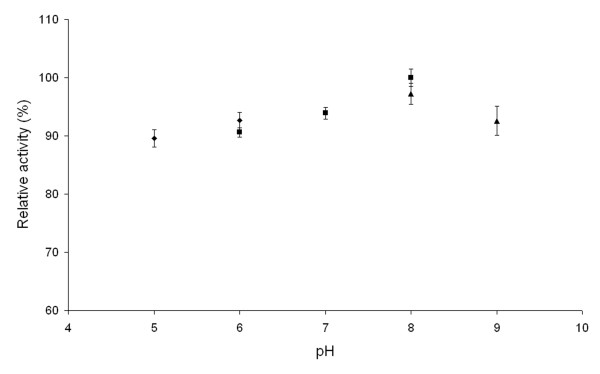
**Effect of pH on activity of recombinant*Arthrobacter*sp. 22c L-arabinose isomerase.** Reaction mixtures containing 0.2 mg mL^-1^*Arthrobacter* sp. 22c L-arabinose isomerase and 4% (w/v) D-galactose in 20 mM citrate buffer pH 5–6 (♦), 20 mM potassium phosphate buffer pH 6–8 (■) and 20 mM Tris–HCl buffer pH 8–9 (▴) were incubated at 50°C for 12 h.

In order to determine the optimum pH for recombinant L-arabinose isomerase activity, conversion of D-galactose to D-tagatose was performed at various pH values and at 50°C. The enzyme exhibited maximum activity at pH 8.0 and maintained over 90% of maximum activity in a pH range of 5 to 9 (Figure 4).

For examination of the metal ion requirements, the purified 22cAI was treated with 10 mM EDTA in order to remove metal ions, dialyzed against 20 mM HEPES pH 7.0 and then assayed in the presence of Ca^2+^, Co^2+^, Mg^2+^, Mn^2+^ and Ni^2+^ ions. No activity loss was observed after treatment with EDTA for 12 h. The enzyme was marginally activated by Mg^2+^, Mn^2+^ and Ca^2+^ ions, and slightly inhibited by Co^2+^ and Ni^2+^ ions (Table 3).

**Table 3:** **Effects of metal ions on*Arthrobacter*sp. 22c L-arabinose isomerase activity**

**Concentration of metal ions**		**Relative activity (%)**	
	**1 mM**	**5 mM**	**10 mM**
None	100	100	100
Ca^2+^	109 ± 1.8	105 ± 2.2	94 ± 1.2
Co^2+^	94 ± 1.5	90 ± 0.2	82 ± 0.1
Mg^2+^	110 ± 0.3	109 ± 0.1	110 ± 1.0
Mn^2+^	106 ± 6.5	105 ± 0.2	105 ± 0.1
Ni^2+^	81 ± 0.4	76 ± 0.3	70 ± 0.4

The kinetic parameters of *Arthrobacter* sp. 22c L-arabinose isomerase for D-galactose as a substrate were determined at 50°C and pH 7.0. The *K*_m_, *V*_max_ and catalytic efficiency (*k*_cat_/*K*_m_) were 119 mM, 0.31 U mg^-1^ and 0.14 mM^-1^ min^-1^, respectively.

**Figure 5:**
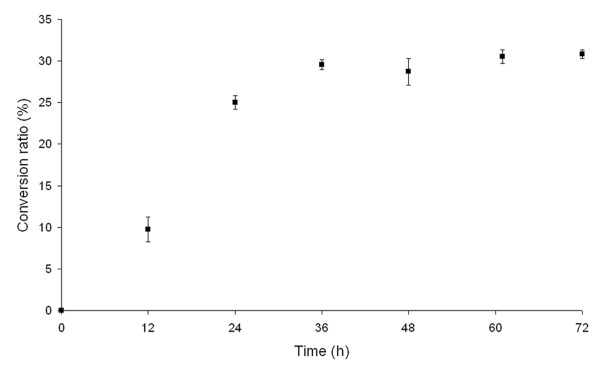
**Time course of D-tagatose production during*Arthrobacter*sp. 22c L-AI-catalyzed isomerization of D-galactose.** Reaction mixtures containing 0.2 mg mL^-1^*Arthrobacter* sp. 22c L-arabinose isomerase and 4% (w/v) D-galactose in 20 mM potassium phosphate buffer pH 7.0 were incubated at 50°C.

Production of D-tagatose from D-galactose by *Arthrobacter* sp. 22c L-arabinose isomerase demonstrated that the conversion of D-galactose to D-tagatose reached equilibrium after 36 h incubation (pH 7.0, 50°C), with a conversion ratio of 30% (Figure 5).

### Construction and characterization of a recombinant *Pichia pastoris* strain secreting β-D-galactosidase *Arthrobacter chlorophenolicus*

**Figure 6:**
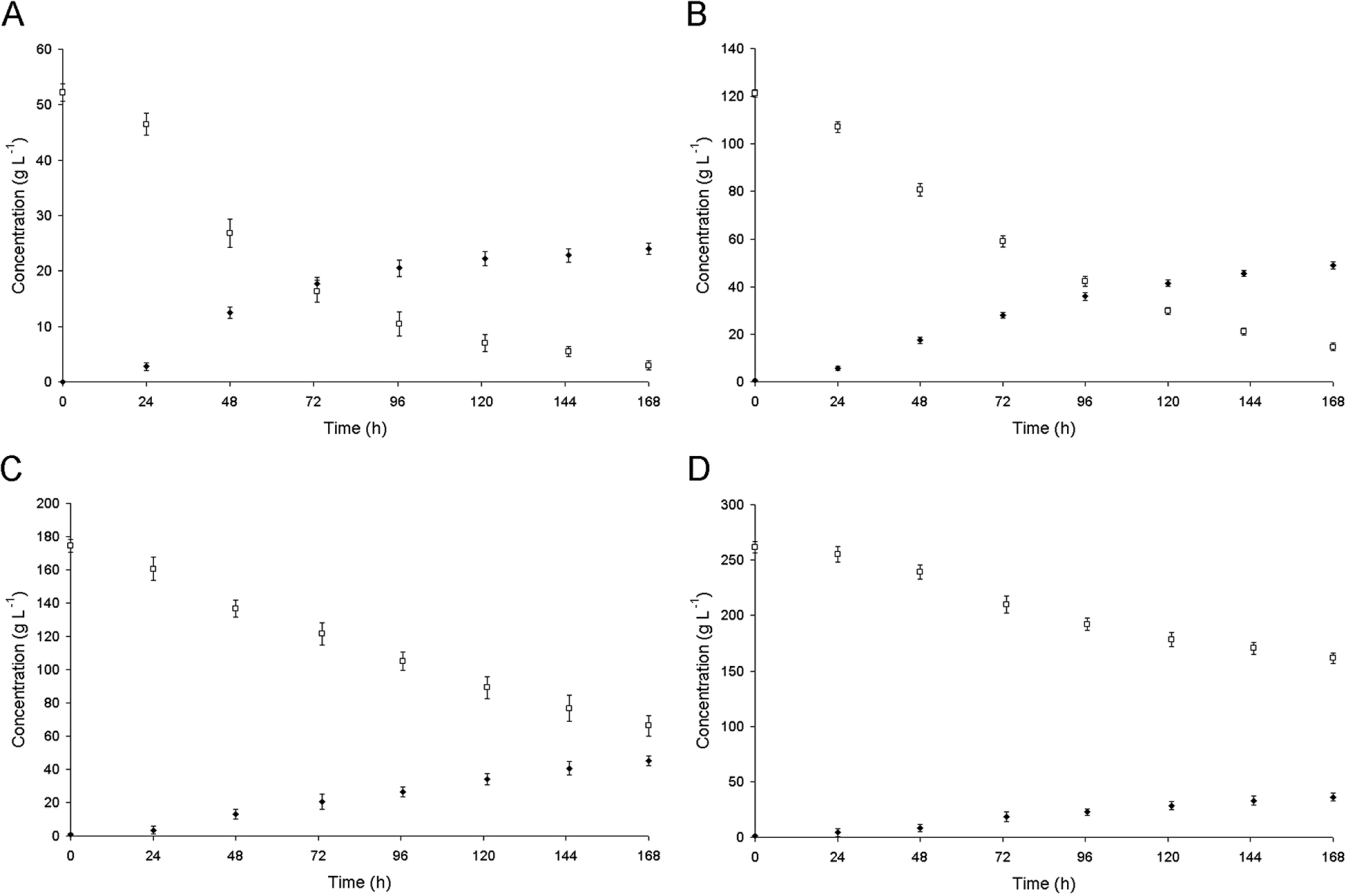
**Time course of D-galactose production from whey permeate using*P. pastoris*strain secreting β-D-galactosidase*A. chlorophenolicus*: (□) lactose, (♦) D-galactose.** Panel **A**: whey permeate containing 60 g L^-1^ lactose before inoculation with recombinant *P. pastoris* strain. Panel **B**: whey permeate containing 140 g L^-1^ lactose before inoculation with recombinant *P. pastoris* strain. Panel **C**: whey permeate containing 200 g L^-1^ lactose before inoculation with recombinant *P. pastoris* strain. Panel **D**: whey permeate containing 300 g L^-1^ lactose before inoculation with recombinant *P. pastoris* strain.

In order to construct a recombinant *Pichia pastoris* strain secreting β-D-galactosidase *Arthrobacter chlorophenolicus*, the gene encoding β-D-galactosidase was cloned under the control of a constitutive glyceraldehyde 3-phosphate dehydrogenase (*GAP*) promoter in the form of translational fusion with the *Saccharomyces cerevisiae* α-factor leader sequence. The recombinant pGAPZα-β-galAch plasmid was then used to transform the *P. pastoris* competent cells. The recombinant yeast strain obtained was able to grow in a medium containing lactose as the sole carbon source. Moreover, growing the recombinant *P. pastoris* strain secreting β-D-galactosidase in the whey permeate resulted in the hydrolysis of lactose and utilization of D-glucose, while D-galactose was accumulated in the medium. The efficient hydrolysis of lactose, at approximately 90% and production of D-galactose was achieved in a whey permeate containing up to 120 g L^-1^ lactose (Figure 6 A and B), after 168 h at 30°C. However, the higher concentrations of lactose in the cultivation medium resulted in lower yields of hydrolysis (Figure 6 C and D), and the formation of galactooligosaccharides.

### Production of D-tagatose using the *Pichia pastoris* strain secreting β-D-galactosidase *Arthrobacter chlorophenolicus* and the recombinant *Arthrobacter* sp. 22c L-arabinose isomerase

**Figure 7:**
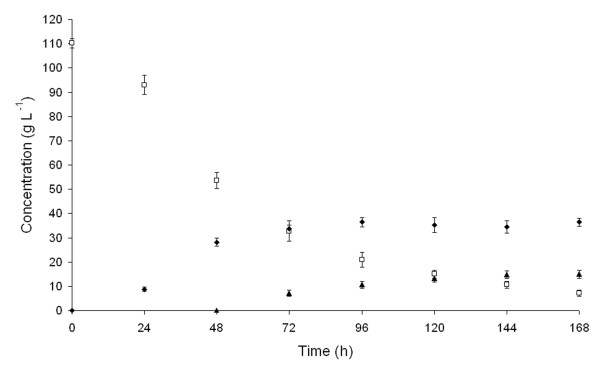
**Time course of D-tagatose production from whey permeate using*P. pastoris*strain secreting β-D-galactosidase*A. chlorophenolicus*and recombinant*Arthrobacter*sp. 22c L-arabinose isomerase: (□) lactose, (♦) D-galactose, (▴) D-tagatose.**

D-Tagatose was obtained by simultaneous production and isomerization of D-galactose. The recombinant *P. pastoris* strain secreting β-D-galactosidase *A. chlorophenolicus* was grown in a whey permeate containing 110 g L^-1^ lactose at 30°C with agitation. After 48 h, when the lactose was partially hydrolyzed and D-galactose was present in the medium, at a quantity of 28 g L^-1^, the purified L-arabinose isomerase *Arthrobacter* sp. 22c was added to an amount of 0.2 mg mL^-1^ and cultivation was continued for 120 h. As shown in Figure 7, after 144 h of cultivation, the conversion of D-galactose into D-tagatose had reached equilibrium and the concentrations of lactose, D-galactose and D-tagatose in the medium were 10.6, 34.4 and 14.8 g L^-1^, respectively, corresponding to a 90% yield of lactose hydrolysis and 30% yield of D-galactose bioconversion.

## Discussion

The article has described the cloning, purification and characterization of L-arabinose isomerase from psychrotolerant Antarctic bacterium *Arthrobacter* sp. 22c. This enzyme exists in the solution as a hexamer, similarly to L-arabinose isomerases from mesophilic bacteria *E. coli* or *L. plantarum* NC8, although most of the L-AIs characterized to date are tetramers (Table 4).

**Table 4:** Biochemical properties of L-arabinose isomerases from various microorganisms

**Organism**	**Molecular mass (kDa)**	**Oligomeric state**	**T**_**opt**_**(°C)**	**pH**_**opt**_	**Metal ion requirement**	**References**
*Arthrobacter* sp. 22c	335 (55.2)^a^	6	47-52	5.0-9.0	Not required	This study
*Lactobacillus sakei* 23 K	220 (53.7)^a^	4	30-40	5.0-7.0	Mn^2+^, Mg^2+^	32
*Escherichia coli*	362 (56.0)^a^	6	30	8.0	Fe^2+^, Mn^2+^	18, 37
*Bacillus halodurans*	224 (56.3)^a^	4	50	7.5-8.0	Not required	19
*Lactobacillus plantarum* NC8	342 (53.6)^a^	6	60	7.5	Mn^2+^, Co^2+^	20
*Thermus* sp. IM6501	(56.0)^a^	NR	60	8.5	Mn^2+^	27
*Lactobacillus fermentum* CGMCC2921	(53.4)^a^	NR	65	6.5	Mn^2+^, Co^2+^	21
*Bacillus stearothermophilus* IAM 11001	(55.9)^a^	NR	65	7.5	Mn^2+^	24
*Alicyclobacillus acidocaldarius*	224 (56.0)^a^	4	65	6.0-6.5	Mn^2+^, Co^2+^, Mg^2+^	25
*Thermoanaerobacter mathranii*	220 (52.8)^a^	4	65	8.0	Mn^2+^	29
*Geobacillus stearothermophilus*	224 (56.1)^a^	4	70	7.0-7.5	Mn^2+^, Co^2+^, Mg^2+^	19
*Geobacillus thermodenitrificans*	230 (56.1)^a^	4	70	8.5	Mn^2+^	22
*Acidothermus cellulolytics* ATCC 43068	(54.8)^a^	NR	75	7.5	Mn^2+^, Co^2+^	28
*Bacillus stearothermophilus* US100	225 (56.2)^a^	4	80	7.5-8.0	Mn^2+b^, Co^2+b^	23
*Thermotoga neapolitana*	(56.7)^a^	NR	85	7.0	Mn^2+^, Co^2+^	30
*Thermotoga maritima*	230 (56.7)^a^	4	90	7.5	Mn^2+^, Co^2+^	31
*Anoxybacillus flavithermus* TC-06	(55.9)^a^	NR	95	9.5-10.5	Ni^2+^	26

NR not reported; ^a^ molecular mass of the monomer, calculated from the amino acid sequence; ^b^ metal ions required only for thermostability [37].

The *Arthrobacter* sp. 22c L-arabinose isomerase is optimally active at relatively low temperatures ranging from 47 to 52°C and it exhibits high activity at an acidic pH. These features make this it attractive for the conversion of D-galactose into D-tagatose, since there is no formation of undesirable by-products and it allows energy to be saved. Only one L-arabinose isomerase, which was isolated from the psychrotolerant bacterium *L. sakei* 23 K, has similar properties (Table 4). However, the latter enzyme needs Mn^2+^ or Mg^2+^ ions for maximum activity and stability, whereas the *Arthrobacter* sp. 22c L-arabinose isomerase does not require the addition of divalent metal ions to the reaction mixture for high activity and stability. The main disadvantage of 22cAI is the relatively low D-galactose bioconversion rate of 30% as compared to others L-arabinose isomerases, especially those which are thermostable (Table 5). However, the authors suppose that D-tagatose production yield can be increased by mutagenesis of 22cAI, as has been achieved in the case of the L-arabinose isomerase from *G. thermodenitrificans*[40]. 

**Table 5:** Bioconversion of D-galactose into D-tagatose using L-arabinose isomerases from various microorganisms

**Organism**	**Conversion yield (%)**	**Conversion conditions**	**References**
*Arthrobacter* sp. 22c	30	50°C, 36 h	This study
*Lactobacillus sakei* 23 K	36	40°C, 7 h	32
*Escherichia coli*	34	35°C, 168 h	38
*Bacillus halodurans*	negligible	NR	19
*Lactobacillus plantarum* NC8	30	60°C, 6 h	20
*Thermus* sp. IM6501	54	60°C, 3 days	27
*Lactobacillus fermentum* CGMCC2921	55	65°C, 12 h	21
*Bacillus stearothermophilus* IAM 11001	36	65°C, 12 h	24
*Alicyclobacillus acidocaldarius*	44	60°C, 6 h	25
*Thermoanaerobacter mathranii*	25	65°C, 24 h	29, 39
*Geobacillus stearothermophilus*	38	60°C, 6 h	19
*Geobacillus thermodenitrificans*	46	65°C, 5 h	22
*Acidothermus cellulolytics* ATCC 43068	53	75°C, 12 h	28
*Bacillus stearothermophilus* US100	48	70°C, 7 h	23
*Thermotoga neapolitana*	68	80°C, 20 h	30
*Thermotoga maritima*	56	80°C, 6 h	31
*Anoxybacillus flavithermus* TC-06	60	95°C, 1 h, borate^a^	26

^a^ The addition of borate can promote D-tagatose formation [38,39].

The article also presents the construction and characterization of a recombinant *P. pastoris* strain secreting β-D-galactosidase from psychrotolerant bacterium *A. chlorophenolicus*[41]. *P. pastoris* yeast was selected for this study owing to its inability to metabolize D-galactose. *P. pastoris* lacks the enzymes constituting the Leloir pathway such as galactokinase, galactose-1-phosphate uridylyltransferase and UDP-galactose 4-epimerase (KEGG PATHWAY Database). Moreover, *P. pastoris* is one of the yeasts to have been widely used for the production and secretion of heterologous proteins [42]. The recombinant yeast strain constructed is able to grow in a medium containing lactose as the sole carbon source. During cultivation, the lactose is hydrolyzed by β-D-galactosidase, and D-glucose is metabolized, while D-galactose is accumulated in the medium. Hence, the *P. pastoris* strain secreting β-D-galactosidase has the potential of being an interesting biocatalyst for the industrial production of D-galactose. Although Grosová et al. have described a method of D-galactose production from lactose by means of simultaneous saccharification and fermentation using β-D-galactosidase from *Kluyveromyces lactis* and *S. cerevisiae* yeast strain entrapped in poly(vinylalcohol) hydrogel [43], the method described in this article would appear to be both less costly and less complicated, since the production and purification of the enzyme have been omitted, as have the immobilization processes.

Moreover, in combination with the *Arthrobacter* sp. 22c L-arabinose isomerase, the *P. pastoris* strain secreting β-D-galactosidase *A. chlorophenolicus* provides an innovative method of producing D-tagatose directly from lactose-containing feedstock. The process allows the simultaneous hydrolysis of disaccharide, utilization of D-glucose and isomerization of D-galactose, and thus reduces both the time and the cost of D-tagatose preparation, in comparison to the processes involving the hydrolysis of lactose and separation of D-galactose from a mixture of D-glucose and D-galactose prior to isomerization [17,33,34].

## Conclusions

The new L-arabinose isomerase from psychrotolerant *Arthrobacter* sp. 22c obtained and characterized in this study has interesting properties. It exhibits optimum activity towards D-galactose at relatively low temperatures ranging from 47 to 52°C and in a broad pH range of between 5 and 9. Moreover, this enzyme does not require the addition of divalent metal ions such as Mn^2+^ or Co^2+^ to the reaction mixture for high activity and stability, unlike most of the L-arabinose isomerases described to date.

In this study, a recombinant *P. pastoris* strain secreting *A. chlorophenolicus* β-D-galactosidase, which can be used in the production of D-galactose from lactose-containing materials was also constructed.

Furthermore, the research has produced an innovative approach to the production of D-tagatose from lactose by means of the simultaneous hydrolysis of disaccharide, utilization of D-glucose and isomerization of D-galactose using a recombinant yeast strain secreting β-D-galactosidase and a recombinant cold-adapted L-arabinose isomerase.

## Methods

### Selection of bacterial strains exhibiting L-arabinose isomerase activity

Twenty bacterial strains of Antarctic microorganisms isolated from soil samples collected in the neighbourhood of the Henryk Arctowski Polish Antarctic Station on King George Island (Southern Shetlands, 62°10’S, 58°28’W) and held in the collections of the Department of Microbiology at Gdańsk University of Technology collection were grown in an LBS medium (0.5% peptone K, 0.25% yeast extract, 1% marine salt) supplemented with 1% L-arabinose at 25°C for 48 h with agitation (200 rpm). The cells were then harvested by centrifugation (10,000x*g*, 20 min, 4°C) and the cell pellets were washed with a 20 mM potassium phosphate buffer with a pH of 7.0. Cell lysis was achieved by grinding with aluminium oxide (Sigma, USA). After grinding, a 20 mM potassium phosphate buffer with a pH of 7.0 was added and the samples were centrifuged (10,000x*g*, 30 min, 4°C) to remove cell debris and aluminium oxide. The supernatants thus obtained were mixed with a 5% (w/v) D-galactose solution in 20 mM potassium phosphate buffer with a pH of 7.0 in a ratio of 1 to 9 and incubated at 25°C for 24 h. An acetonitrile was then added to the samples at a ratio of 1 to 1 and the quantities of D-galactose and D-tagatose were determined by HPLC, using an LiChrospher 100 NH_2_ column (Merck, Germany), 80% (v/v) acetonitrile as a mobile phase and the Agilent 1200 Series Refractive Index Detector.

### Identification of the 22c strain

Genomic DNA from strain 22c, was used as a template to amplify the 16S rDNA gene with the primers 16SFor 5’ AGAGTTTGATCCTGGCTCAG 3’ and 16SRev 5’ ACGGCTACCTTGTTACGACTT 3’. A PCR reaction was performed in a mixture containing 0.2 μM of each primer, 0.2 μg of genomic DNA, 200 μM of each dNTP, and 1 U of DNA polymerase *Hypernova* (DNA-Gdańsk II, Poland) in 1 x PCR buffer (10 mM Tris–HCl pH 8.8, 50 mM KCl, 3 mM MgCl_2_, 0.15% Triton X-100). The reaction mixture was incubated for 3 min at 95°C, followed by 30 cycles at 95°C for 1 min, 52°C for 1 min, 72°C for 1.5 min, and a final incubation of 5 min at 72°C, using a Mastercycler Gradient (Eppendorf, Germany). The PCR product was purified from an agarose gel band using a DNA Gel-Out kit (A&A Biotechnology, Poland), cloned into a pJET1.2/blunt vector (Fermentas, Lithuania) and sequenced (Genomed, Poland).

### Isolation and sequencing of the *araA* gene from *Arthrobacter* sp. 22c

The gene encoding the L-arabinose isomerase from *Arthrobacter* sp. 22c was isolated using the PCR technique. In order to obtain a partial sequence of the *araA* gene from *Arthrobacter* sp. 22c, sequences encoding genes of the arabinose operons (*araB*, *araD* and *araA*) of *Arthrobacter chlorophenolicus* A6 [GenBank: NC_011886], *Arthrobacter aurescens* TC1 [GenBank: NC_008711] and *Arthrobacter* sp. FB24 [GenBank: NC_008541], obtained from the GenBank database, were aligned using the ClustalX program, version 1.8. On the basis of the alignment, degenerated primers araDF 5’ CTGATGCARAACCACGGCCCSTTCACCATCGGC 3’ and araAR 5’ GTCTTCCTTGCCGCCRATGCCSAGCGGGTG 3’ were designed and synthesized. The PCR reaction was performed in a mixture containing 0.2 μM of each primer, 0.2 μg of *Arthrobacter* sp. 22c genomic DNA, 200 μM of each dNTP, and 1 U of DNA polymerase *Hypernova* (DNA-Gdańsk II, Poland) in 1 x PCR buffer (10 mM Tris–HCl pH 8.8, 50 mM KCl, 3 mM MgCl_2_, 0.15% Triton X-100). The reaction mixture was incubated for 3 min at 95°C, followed by 30 cycles at 95°C for 1 min, 72°C for 2.5 min, and a final incubation of 5 min at 72°C. The PCR product thus obtained was then purified, cloned into the pJET1.2/blunt vector (Fermentas, Lithuania) and sequenced. The GenomeWalker™ Universal Kit (Clontech Laboratories, USA) was then used to obtain the 3’ end of *Arthrobacter* sp. 22c *araA* gene. The *Arthrobacter* sp. 22c genomic DNA was first digested with the *Pvu*II restriction endonuclease provided in the kit and ligated to the GenomeWalker Adaptor. Two PCR amplifications were then performed. The primary reaction was carried out using adaptor-ligated genomic DNA fragments as a template and two primers; the outer adaptor primer (AP1) provided in the kit and 22c out primer 5’ GCCTCGCTGATGGAGGATTACACCTATGACCTGAC 3’, designed on the basis of the partial sequence of *Arthrobacter* sp. 22c *araA* gene previously obtained. The reaction mixture also contained 200 μM dNTPs and 1 U of DNA polymerase Marathon (A&A Biotechnology, Poland) in 1 x Marathon buffer. DNA amplification was performed using the following parameters: (94°C – 0.5 min, 72°C – 3 min) 7 cycles, (94°C – 0.5 min, 67°C – 3 min) 32 cycles and 67°C for 7 min after the final cycle. The primary PCR mixture was then used as a template for a secondary PCR with the nested adaptor primer (AP2) provided in the kit and 22c32c in primer 5’ GATCCTSGGCGCGCACATGCTKGAGG 3’. The nested PCR mixture also contained 200 μM dNTPs and 1 U of DNA polymerase Marathon in 1 x Marathon buffer. DNA amplification was performed using the following parameters: (94°C – 0.5 min, 72°C – 3 min) 5 cycles, (94°C – 0.5 min, 67°C – 3 min) 20 cycles and 67°C for 7 min after the final cycle. In the third step the nested PCR product was purified from an agarose gel band, cloned into the pJET1.2/blunt vector and sequenced. Following this, two fragments of *Arthrobacter* sp. 22c genomic DNA sequences were alignment and the full sequence of the *Arthrobacter* sp. 22c *araA* gene was obtained.

### Construction of the *E. coli* expression system for the production of *Arthrobacter* sp. 22c L-arabinose isomerase

On the basis of the known sequence of the *araA* gene from *Arthrobacter* sp. 22c (GenBank Accession No. JN642528), the specific primers for PCR amplification were designed and synthesized. The gene was amplified using forward primer F22cNdemut 5’ ATACAT**ATG**AGCAAAGCCTATGAATCCAAGGA 3’, and reverse primer R22cHind 5’ CTGAAGC**TTA**CAGGCCCTGCGCCAGACGGTAGTACGCCTGG 3’ (containing *Nde*I and *Hin*dIII recognition sites, underlined). The start and stop codons are given in bold. The PCR reaction mixture contained 0.2 μM of each primer, 0.2 μg of *Arthrobacter* sp. 22c genomic DNA, 200 μM of each dNTP, and 1 U of DNA polymerase *Hypernova* (DNA-Gdańsk II, Poland) in 1 x PCR buffer (10 mM Tris–HCl pH 8.8, 50 mM KCl, 3 mM MgCl_2_, 0.15% Triton X-100). The reaction mixture was incubated for 3 min at 95°C, followed by 30 cycles at 95°C for 1 min, 60°C for 1 min, 72°C for 1.5 min and a final incubation of 5 min at 72°C using a Mastercycler Gradient (Eppendorf, Germany). The PCR product was then purified from an agarose gel band using a DNA Gel-Out kit (A&A Biotechnology, Poland), digested with *Nde*I and *Hin*dIII endonucleases (Fermentas, Lithuania) and cloned into a pET-30 Ek/LIC vector (Novagen, England) digested with the same restriction enzymes. The resulting recombinant plasmid pET30araA22c containing the *Arthrobacter* sp. 22c L-arabinose isomerase gene under the control of the T7 promoter was used to transform chemically competent *E. coli* BL21(DE3)pLysS cells (Novagen, England).

### Production and purification of the recombinant *Arthrobacter* sp. 22c L-arabinose isomerase

Expression of the *Arthrobacter* sp. 22c L-arabinose isomerase gene was performed in the *E. coli* BL21(DE3)pLysS cells carrying the pET30araA22c plasmid. The cells were grown at 30°C in 1 litre of LB medium (1% peptone K, 0.5% yeast extract, 1% NaCl) containing 25 μg mL^-1^ kanamycin and 34 μg mL^-1^ chloramphenicol to OD_600_ of 0.4. 1 M isopropyl β-D-1-thiogalactopyranoside (IPTG) was then added to the final concentration of 1 mM and cultivation was continued for 6 h. The culture was then centrifuged (5,000x*g*, 10 min, 4°C), the cell pellet was re-suspended in 50 mL of buffer A (20 mM potassium phosphate buffer pH 6.0 containing 50 mM KCl), and the cells were disrupted by sonication. After centrifugation (10,000x*g*, 30 min, 4°C), the supernatant was applied onto Fractogel® EMD DEAE weak anion exchanger purchased from Merck (Germany) (60 mL column) equilibrated with four volumes of buffer A. The column was washed with three volumes of buffer A and the recombinant L-arabinose isomerase was eluted with a linear gradient of potassium chloride (50–1500 mM) in the same buffer (four volumes of the column). Fractions containing L-arabinose isomerase were pooled, dialyzed against buffer A and loaded onto Fractogel® EMD TMAE (Merck) strong anion exchanger. The 40 mL column had previously been equilibrated with four volumes of buffer A and the elution was performed with a linear gradient of potassium chloride (50–1250 mM) in the same buffer (four volumes of the column). Fractions containing *Arthrobacter* sp. 22c L-arabinose isomerase were pooled, dialyzed against 20 mM potassium phosphate buffer pH 7.0 and stored at 4°C until used. The concentration of purified protein was determined by the Bradford method [44] using bovine serum albumin (BSA) as a standard.

### Characterization of the *Arthrobacter* sp. 22c L-arabinose isomerase

The molecular mass of the native *Arthrobacter* sp. 22c L-arabinose isomerase was estimated by gel filtration using bovine thyroglobulin (669 kDa), apoferritin from horse spleen (443 kDa), β-amylase from sweet potato (200 kDa), alcohol dehydrogenase from yeast (150 kDa), bovine serum albumin (66 kDa) and carbonic anhydrase from bovine erythrocytes (29 kDa) purchased from Sigma (USA) as standards. The purified enzyme was loaded onto a Superdex™ 200 10/300 GL column (Amersham Biosciences AB, Sweden) and the elution was performed using 20 mM potassium phosphate buffer pH 7.0 containing 150 mM KCl.

The activity of purified *Arthrobacter* sp. 22c L-arabinose isomerase was determined using D-galactose as a substrate. The isomerization reaction was halted by the addition of acetonitrile (1:1) and the quantity of D-tagatose was determined by HPLC using a LiChrospher 100 NH_2_ column, 80% (v/v) acetonitrile as a mobile phase and a refractive index detector. One unit of L-arabinose isomerase activity was defined as being the quantity of enzyme catalysing the formation of 1 μmol D-tagatose per min at 50°C and pH 7.0.

To determine the effect of temperature on the L-arabinose isomerase’s activity, the enzyme (0.2 mg mL^-1^) was incubated in 20 mM potassium phosphate buffer pH 7.0 containing 4% (w/v) substrate at 25 to 65°C for 12 h.

For pH-activity studies, isomerization reactions were performed in 20 mM citrate buffer pH 5 to 6, 20 mM potassium phosphate buffer pH 6 to 8 and 20 mM Tris–HCl buffer pH 8 to 9 at 50°C.

The effects of various metal ions on recombinant enzyme activity were measured by assaying the enzyme in 20 mM HEPES pH 7.0 containing 1, 5 and 10 mM CaCl_2_·2H_2_O, CoCl_2_·6H_2_O, MgCl_2_·6H_2_O, MnCl_2_·4H_2_O or NiCl_2_·6H_2_O at 50°C.

For the thermal stability studies, the enzyme was incubated at various temperatures for different periods of time and the residual L-arabinose isomerase activity was then measured at 50°C and pH 7.0.

To determine the kinetic parameters, the isomerization reactions were performed in 20 mM potassium phosphate buffer pH 7.0 containing 50–500 mM D-galactose at 50°C for 30 min. The *K*_m_ and *V*_max_ values were obtained using the Lineweaver-Burk equation.

Analysis of the isomerization of D-galactose to D-tagatose by the *Arthrobacter* sp. 22c L-arabinose isomerase was performed in a mixture containing 4% (w/v) D-galactose and 0.2 mg mL^-1^ L-arabinose isomerase in 20 mM potassium phosphate buffer pH 7.0. The reaction mixture was incubated at 50°C for 72 h. After each 12-hour period, 1 mL samples were collected and the isomerization reaction was halted by the addition of acetonitrile. D-Tagatose and D-galactose concentrations were determined by HPLC.

### Construction of the recombinant *P. pastoris* strain secreting β-D-galactosidase *A. chlorophenolicus*

*Arthrobacter chlorophenolicus* (DSM 12829) was purchased from Deutsche Sammlung von Mikroorganismen und Zellkulturen (Germany). The primers used for PCR amplification of the β-D-galactosidase gene were designed on the basis of the known *A. chlorophenolicus* β-D-galactosidase gene sequence [GenBank: NC_011886 (3472060.3474075)]. These primers were FAchBSal 5’ ATAGTCGACAAAAGA**ATG**GCAACGCAGGAAATTAACCGTCCG 3’ containing *Sal*I recognition site (underlined) and RAchBXbH 5’CTGAAGCTTCTAGA**CTA**GCCCTCCCTGATCACCGCAATGC 3’ containing *Xba*I and *Hin*dIII recognition sites (underlined). The start and stop codons are given in bold. The PCR reaction mixture contained 0.2 μM of each primer, 0.2 μg of *A. chlorophenolicus* genomic DNA, 200 μM of each dNTP, and 1 U of DNA polymerase *Hypernova* (DNA-Gdańsk II, Poland) in 1 x PCR buffer (10 mM Tris–HCl pH 8.8, 50 mM KCl, 3 mM MgCl_2_, 0.15% Triton X-100). The reaction mixture was incubated for 3 min at 95°C, followed by 30 cycles at 95°C for 1 min, 68°C for 1 min, 72°C for 2 min and a final incubation of 5 min at 72°C using a Mastercycler Gradient (Eppendorf, Germany). The PCR product thus obtained was purified from an agarose gel band, digested with *Sal*I and *Xba*I endonucleases (Fermentas, Lithuania) and cloned into a pGAPZα B vector (Invitrogen, USA) previously digested with *Xho*I and *Xba*I restriction enzymes. The *Sal*I and *Xho*I endonucleases leave compatible protruding DNA ends. The resulting recombinant plasmid pGAPZα-β-galAch containing the *A. chlorophenolicus* β-D-galactosidase gene was then linearized by *Xma*JI endonuclease and used to transform *P. pastoris* GS115 competent cells using *Pichia* EasyComp™ Transformation Kit (Invitrogen). The secretion of *A. chlorophenolicus* β-D-galactosidase by the recombinant *P. pastoris* strain harbouring pGAPZα-β-galAch plasmid was confirmed in the reaction with *o*-nitrophenyl β-D-galactopyranoside (ONPG). The recombinant yeast strain was grown in a YPD medium (2% peptone K, 1% yeast extract, 2% D-glucose) at 30°C for 72 h and the enzyme activity was then determined in the medium obtained after centrifugation of the culture (5,000×*g*, 10 min, 4°C). 0.25 mL of postcultivation medium was added to 0.75 mL of ONPG solution (1 mg mL^-1^ in 20 mM potassium phosphate buffer pH 7.0) and the mixture was incubated at 30°C for 15 min. The hydrolysis reaction was then halted by the addition of 0.5 mL 1 M Na_2_CO_3_ and the absorbance of the mixture was measured at 405 nm. The reference sample contained fresh YPD medium instead of postcultivation medium.

### Production of D-galactose using the recombinant *P. pastoris* strain secreting β-D-galactosidase *A. chlorophenolicus*

The recombinant *P. pastoris* strain harbouring the pGAPZα-β-galAch plasmid was used for D-galactose production from whey permeate. The whey permeate powder, containing 80% (w/w) lactose, was purchased from the OSTROWIA Mazovian Dairy Co-operative (Poland). The recombinant yeast strain was grown in the YPD medium (2% peptone K, 1% yeast extract, 2% D-glucose), at 30°C for 30 h with agitation (250 rpm). This culture was used to inoculate (15% v/v) whey permeate solutions containing 60–300 g L^-1^ lactose. The cultures were then grown for 168 h at 30°C. After each 24-hour period, 1 mL samples were collected and the hydrolysis of lactose catalyzed by the secreted *A. chlorophenolicus* β-D-galactosidase was halted by the addition of 17 μL of 20% H_2_SO_4_. The quantities of lactose and D-galactose in the supernatants were determined by HPLC using an Aminex HPX-87H column (Bio-Rad, USA), 5 mM H_2_SO_4_ as a mobile phase and an Agilent 1200 Series Refractive Index Detector.

### Production of D-tagatose using the recombinant *Pichia pastoris* strain secreting β-D-galactosidase *A. chlorophenolicus* and the recombinant L-arabinose isomerase from *Arthrobacter* sp. 22c

To produce D-tagatose, a whey permeate solution containing 130 g L^-1^ lactose was inoculated with 30 h culture of the recombinant *P. pastoris* strain harbouring pGAPZα-β-galAch plasmid (15% v/v) and incubated for 48 h at 30°C with agitation (250 rpm). 0.2 mg mL^-1^ of the purified *Arthrobacter* sp. 22c L-arabinose isomerase was then added and cultivation was continued for 120 h. After each 24-hour period, 1 mL samples were taken and centrifuged (10,000x*g*, 10 min, 4°C). The enzymatic reactions were halted by heating for 10 min at 95°C. The samples were then cooled and filtered through a 0.45 μm filter. The quantities of lactose, D-galactose and D-tagatose were determined by HPLC using an Aminex HPX-87C column (Bio-Rad, USA), deionized water as a mobile phase and an Agilent 1200 Series Refractive Index Detector.

All the measurements were taken and the experiments carried out in triplicate.

### Nucleotide sequences accession numbers

The 16S rDNA and L-arabinose isomerase gene sequences reported in this article have been deposited in the GenBank database and assigned Accession Nos. JN642527 and JN642528, respectively.

## Abbreviations

L-AI: L-Arabinose isomerase; 22cAI: L-Arabinose isomerase from *Arthrobacter* sp. 22c; EDTA: Ethylenediaminetetraacetic acid; GAP: Glyceraldehyde 3-phosphate dehydrogenase; HEPES: N-(2-Hydroxyethyl)piperazine-N’-(2-ethanesulfonic acid); HPLC: High performance liquid chromatography; IPTG: Isopropyl β-F-1-thiogalactopyranoside; ONPG: *o*-Nitrophenyl β-D-galactopyranoside.

## Competing interest

The authors declare that they have no competing interests.

## Authors’ contributions

MW designed the study, performed the experiments and drafted the manuscript, JK coordinated the study and revised the manuscript. Both authors have read and approved the final version of manuscript.
